# 5-[4-(1*H*-Imidazol-1-yl)phen­yl]-1*H*-tetra­zole

**DOI:** 10.1107/S1600536812013670

**Published:** 2012-04-04

**Authors:** Shao-Wei Tong, Wen-Dong Song, Shi-Jie Li, Dong-Liang Miao, Jing-Bo An

**Affiliations:** a College of Food Science and Technology, Guangdong Ocean University, Zhanjiang 524088, People’s Republic of China; b College of Science, Guangdong Ocean University, Zhanjiang 524088, People’s Republic of China; c School of Environment Science and Engineering, Donghua University, Shanghai 200051, People’s Republic of China

## Abstract

In the title compound, C_10_H_8_N_6_, the tetra­zole and benzene rings are close to being coplanar [dihedral angle = 9.90 (16)°], but the imidazole ring is rotated 37.18 (09)° out of the benzene plane. In the crystal, mol­ecules are connected through tetra­zole–imidazole N—H⋯N hydrogen bonds, giving rise to zigzag chains, which extend along [010].

## Related literature

For our previous work based on the imidazole derivatives as ligands, see: Li *et al.* (2010 ▶); Tong *et al.* (2011 ▶); Tong *et al.*, (2012 ▶). For related structures, see: Huang *et al.* (2009 ▶); Cheng (2011 ▶).
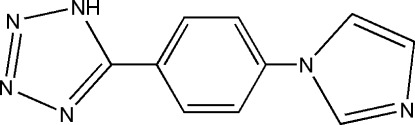


## Experimental

### 

#### Crystal data

C_10_H_8_N_6_
*M* *_r_* = 212.22
Monoclinic, 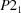

*a* = 3.7219 (12) Å
*b* = 16.429 (5) Å
*c* = 7.791 (2) Å
β = 97.167 (6)°
*V* = 472.6 (3) Å^3^
*Z* = 2
Mo *K*α radiation
μ = 0.10 mm^−1^
*T* = 296 K
0.30 × 0.20 × 0.15 mm

#### Data collection

Bruker SMART CCD area-detector diffractometer
Absorption correction: multi-scan (*SADABS*; Bruker, 2007 ▶) *T*_min_ = 0.971, *T*_max_ = 0.985
3477 measured reflections
1421 independent reflections
1239 reflections with *I* > 2σ(*I*)
*R*_int_ = 0.038

#### Refinement

*R*[*F* ^2^ > 2σ(*F* ^2^)] = 0.039
*wR*(*F* ^2^) = 0.120
*S* = 1.35
1421 reflections
145 parameters
1 restraint
H-atom parameters constrained
Δρ_max_ = 0.25 e Å^−3^
Δρ_min_ = −0.31 e Å^−3^

### 

Data collection: *SMART* (Bruker, 2007 ▶); cell refinement: *SAINT* (Bruker, 2007 ▶); data reduction: *SAINT*; program(s) used to solve structure: *SHELXS97* (Sheldrick, 2008 ▶); program(s) used to refine structure: *SHELXL97* (Sheldrick, 2008 ▶); molecular graphics: *SHELXTL* (Sheldrick, 2008 ▶); software used to prepare material for publication: *SHELXTL*.

## Supplementary Material

Crystal structure: contains datablock(s) I, global. DOI: 10.1107/S1600536812013670/zs2194sup1.cif

Structure factors: contains datablock(s) I. DOI: 10.1107/S1600536812013670/zs2194Isup2.hkl

Supplementary material file. DOI: 10.1107/S1600536812013670/zs2194Isup3.cml

Additional supplementary materials:  crystallographic information; 3D view; checkCIF report

## Figures and Tables

**Table 1:** Hydrogen-bond geometry (Å, °)

*D*—H⋯*A*	*D*—H	H⋯*A*	*D*⋯*A*	*D*—H⋯*A*
N3—H3⋯N2^i^	0.86	1.93	2.751 (4)	158

Symmetry code: (i) 
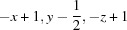
.

**Fig. 1.**
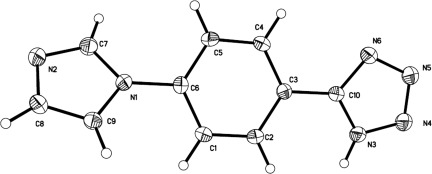
Molecular conformation and atom numbering scheme for the title compound, showing 30% probability displacement ellipsoids.

**Fig. 2.**
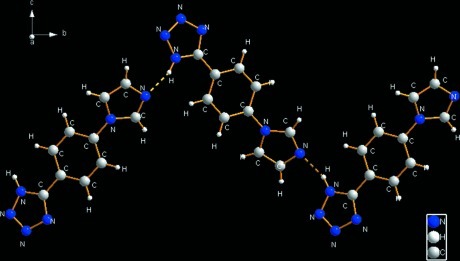
The one-dimensional chain structure extending along [010], with hydrogen bonds shown as dashed lines.

## supplementary crystallographic information

### Comment

The imidazole derivatives can be used to synthesize various types of metal complexes because they contain available *N*-donor sites for coordination. Our research group has shown great interest in metal-organic complexes with imidazole derivatives, e.g. 2-propylimidazole-4,5-dicarboxylic acid (Tong *et al.*, 2011; Li *et al.*, 2010) and 5-[4-imidazol-1-yl)phenyl]tetrazole (1-tetrazole-4-imidazolebenzene) (Tong *et al.*, 2012). In this paper, we report the crystal structure of this ligand from crystals obtained under hydrothermal conditions. As illustrated in Fig. 1, the tetrazole and benzene rings are close to coplanar [dihedral angle, 9.90 (16)°] but the imidazole ring is rotated 37.18 (19)° out of the benzene plane. The molecules are connected into one-dimensional zigzag chains through tetrazole N—H···N_imidazole_ hydrogen bonds (Table 1, Fig. 2). For the structures of complexes with this ligand, see Huang *et al.* (2009) and Cheng (2011).

### Experimental

5-[4-imidazol-1-yl)phenyl]tetrazole (0.2 mmol, 0.043 g) in 12 ml of *N*,*N*-dimethylformamide was sealed in an autoclave equipped with a Teflon liner (25 ml) and then heated at 413 K for 3 days. Crystals of the title compound were obtained by slow evaporation of the solvent at room temperature.

### Refinement

The imidazolyl and phenyl H-atoms and the tetrazole N H-atom were located in a difference-Fourier but were refined as riding with C—H = 0.93 Å or N—H = 0.86 Å and with *U*_iso_(H) = 1.2*U*_eq_(C, N).

### Crystal data

**Table:**

C_10_H_8_N_6_	*F*(000) = 220
*M**_r_* = 212.22	*D*_x_ = 1.491 Mg m^−^^3^
Monoclinic, *P*2_1_	Mo *K*α radiation, λ = 0.71073 Å
Hall symbol: P 2yb	Cell parameters from 1003 reflections
*a* = 3.7219 (12) Å	θ = 2.6–22.3°
*b* = 16.429 (5) Å	µ = 0.10 mm^−^^1^
*c* = 7.791 (2) Å	*T* = 296 K
β = 97.167 (6)°	Block, colorless
*V* = 472.6 (3) Å^3^	0.30 × 0.20 × 0.15 mm
*Z* = 2	

### Data collection

**Table:**

Bruker SMART CCD area-detector diffractometer	1421 independent reflections
Radiation source: fine-focus sealed tube	1239 reflections with *I* > 2σ(*I*)
Graphite monochromator	*R*_int_ = 0.038
φ and ω scans	θ_max_ = 25.0°, θ_min_ = 2.5°
Absorption correction: multi-scan (*SADABS*; Bruker, 2007)	*h* = −4→4
*T*_min_ = 0.971, *T*_max_ = 0.985	*k* = −19→18
3477 measured reflections	*l* = −9→9

### Refinement

**Table:**

Refinement on *F*^2^	Primary atom site location: structure-invariant direct methods
Least-squares matrix: full	Secondary atom site location: difference Fourier map
*R*[*F*^2^ > 2σ(*F*^2^)] = 0.039	Hydrogen site location: inferred from neighbouring sites
*wR*(*F*^2^) = 0.120	H-atom parameters constrained
*S* = 1.35	*w* = 1/[σ^2^(*F*_o_^2^) + (0.051*P*)^2^ + 0.001*P*] where *P* = (*F*_o_^2^ + 2*F*_c_^2^)/3
1421 reflections	(Δ/σ)_max_ < 0.001
145 parameters	Δρ_max_ = 0.25 e Å^−^^3^
1 restraint	Δρ_min_ = −0.31 e Å^−^^3^

### Special details

**Table:**

Geometry. All e.s.d.'s (except the e.s.d. in the dihedral angle between two l.s. planes) are estimated using the full covariance matrix. The cell e.s.d.'s are taken into account individually in the estimation of e.s.d.'s in distances, angles and torsion angles; correlations between e.s.d.'s in cell parameters are only used when they are defined by crystal symmetry. An approximate (isotropic) treatment of cell e.s.d.'s is used for estimating e.s.d.'s involving l.s. planes.
Refinement. Refinement of *F*^2^ against ALL reflections. The weighted *R*-factor *wR* and goodness of fit *S* are based on *F*^2^, conventional *R*-factors *R* are based on *F*, with *F* set to zero for negative *F*^2^. The threshold expression of *F*^2^ > σ(*F*^2^) is used only for calculating *R*-factors(gt) *etc*. and is not relevant to the choice of reflections for refinement. *R*-factors based on *F*^2^ are statistically about twice as large as those based on *F*, and *R*- factors based on ALL data will be even larger.

### Fractional atomic coordinates and isotropic or equivalent isotropic displacement parameters (Å^2^)

**Table:**

	*x*	*y*	*z*	*U*_iso_*/*U*_eq_	
N1	0.9106 (8)	0.45885 (16)	0.5643 (3)	0.0314 (7)	
N2	0.9495 (8)	0.57050 (19)	0.7228 (4)	0.0388 (8)	
N3	0.3005 (9)	0.16887 (18)	0.0321 (4)	0.0370 (8)	
H3	0.2517	0.1475	0.1273	0.044*	
N4	0.2320 (10)	0.13459 (19)	−0.1262 (4)	0.0451 (9)	
N5	0.3436 (11)	0.1857 (2)	−0.2329 (4)	0.0487 (9)	
N6	0.4867 (9)	0.2536 (2)	−0.1484 (4)	0.0422 (8)	
C1	0.6945 (10)	0.3256 (2)	0.4631 (4)	0.0350 (9)	
H1A	0.6956	0.3095	0.5776	0.042*	
C2	0.5860 (10)	0.2723 (2)	0.3310 (4)	0.0352 (9)	
H2A	0.5189	0.2195	0.3563	0.042*	
C3	0.5757 (9)	0.2967 (2)	0.1594 (4)	0.0276 (8)	
C4	0.6843 (9)	0.3746 (2)	0.1250 (4)	0.0326 (8)	
H4A	0.6805	0.3911	0.0106	0.039*	
C5	0.7988 (9)	0.4287 (2)	0.2560 (4)	0.0330 (9)	
H5A	0.8719	0.4810	0.2308	0.040*	
C6	0.8024 (9)	0.4037 (2)	0.4249 (4)	0.0287 (8)	
C7	0.8324 (9)	0.5391 (2)	0.5711 (4)	0.0350 (9)	
H7A	0.7107	0.5684	0.4796	0.042*	
C8	1.1139 (10)	0.5070 (2)	0.8171 (4)	0.0359 (9)	
H8A	1.2250	0.5110	0.9305	0.043*	
C9	1.0919 (10)	0.4384 (2)	0.7229 (4)	0.0338 (8)	
H9A	1.1812	0.3873	0.7578	0.041*	
C10	0.4558 (10)	0.2411 (2)	0.0171 (4)	0.0310 (8)	

### Atomic displacement parameters (Å^2^)

**Table:**

	*U*^11^	*U*^22^	*U*^33^	*U*^12^	*U*^13^	*U*^23^
N1	0.0388 (17)	0.0255 (18)	0.0297 (16)	0.0014 (13)	0.0032 (13)	0.0001 (13)
N2	0.050 (2)	0.0313 (17)	0.0346 (16)	0.0023 (15)	0.0045 (14)	−0.0022 (14)
N3	0.0485 (19)	0.0313 (18)	0.0303 (16)	−0.0028 (15)	0.0015 (14)	0.0012 (13)
N4	0.062 (2)	0.0357 (19)	0.0359 (18)	−0.0056 (17)	0.0009 (15)	−0.0041 (15)
N5	0.074 (3)	0.039 (2)	0.0330 (19)	−0.0032 (18)	0.0052 (18)	−0.0041 (16)
N6	0.059 (2)	0.0367 (18)	0.0318 (16)	−0.0055 (16)	0.0080 (15)	−0.0003 (15)
C1	0.047 (2)	0.032 (2)	0.0268 (19)	0.0006 (18)	0.0065 (16)	0.0046 (15)
C2	0.048 (2)	0.027 (2)	0.0317 (19)	−0.0044 (17)	0.0078 (16)	0.0036 (15)
C3	0.0263 (17)	0.0287 (19)	0.0279 (17)	0.0004 (15)	0.0040 (14)	−0.0004 (15)
C4	0.040 (2)	0.034 (2)	0.0249 (19)	−0.0007 (17)	0.0053 (16)	0.0049 (15)
C5	0.038 (2)	0.028 (2)	0.0338 (19)	−0.0045 (17)	0.0094 (16)	0.0049 (15)
C6	0.0290 (19)	0.0288 (19)	0.0281 (18)	0.0000 (16)	0.0027 (15)	−0.0034 (15)
C7	0.037 (2)	0.033 (2)	0.034 (2)	0.0034 (17)	0.0001 (16)	0.0018 (15)
C8	0.039 (2)	0.040 (2)	0.0278 (17)	0.0008 (18)	0.0023 (15)	0.0000 (17)
C9	0.039 (2)	0.031 (2)	0.0293 (18)	0.0042 (17)	−0.0018 (15)	0.0032 (15)
C10	0.0362 (19)	0.029 (2)	0.0281 (19)	−0.0020 (16)	0.0054 (15)	0.0017 (15)

### Geometric parameters (Å, º)

**Table:**

N1—C7	1.353 (4)	C1—H1A	0.9300
N1—C9	1.374 (4)	C2—C3	1.392 (4)
N1—C6	1.433 (4)	C2—H2A	0.9300
N2—C7	1.313 (4)	C3—C4	1.377 (5)
N2—C8	1.376 (5)	C3—C10	1.463 (4)
N3—C10	1.332 (5)	C4—C5	1.381 (5)
N3—N4	1.351 (4)	C4—H4A	0.9300
N3—H3	0.8600	C5—C6	1.377 (4)
N4—N5	1.286 (5)	C5—H5A	0.9300
N5—N6	1.369 (5)	C7—H7A	0.9300
N6—C10	1.324 (4)	C8—C9	1.342 (5)
C1—C2	1.374 (5)	C8—H8A	0.9300
C1—C6	1.387 (5)	C9—H9A	0.9300
			
C7—N1—C9	106.7 (3)	C3—C4—H4A	119.2
C7—N1—C6	127.2 (3)	C5—C4—H4A	119.2
C9—N1—C6	125.9 (3)	C6—C5—C4	118.7 (3)
C7—N2—C8	105.0 (3)	C6—C5—H5A	120.7
C10—N3—N4	109.0 (3)	C4—C5—H5A	120.7
C10—N3—H3	125.5	C5—C6—C1	120.8 (3)
N4—N3—H3	125.5	C5—C6—N1	120.2 (3)
N5—N4—N3	106.2 (3)	C1—C6—N1	118.9 (3)
N4—N5—N6	111.0 (3)	N2—C7—N1	111.7 (3)
C10—N6—N5	105.4 (3)	N2—C7—H7A	124.2
C2—C1—C6	119.6 (3)	N1—C7—H7A	124.2
C2—C1—H1A	120.2	C9—C8—N2	110.6 (3)
C6—C1—H1A	120.2	C9—C8—H8A	124.7
C1—C2—C3	120.4 (3)	N2—C8—H8A	124.7
C1—C2—H2A	119.8	C8—C9—N1	106.0 (3)
C3—C2—H2A	119.8	C8—C9—H9A	127.0
C4—C3—C2	118.7 (3)	N1—C9—H9A	127.0
C4—C3—C10	120.1 (3)	N6—C10—N3	108.3 (3)
C2—C3—C10	121.1 (3)	N6—C10—C3	125.9 (3)
C3—C4—C5	121.7 (3)	N3—C10—C3	125.8 (3)
			
C10—N3—N4—N5	−0.2 (4)	C9—N1—C6—C1	35.1 (5)
N3—N4—N5—N6	0.2 (4)	C8—N2—C7—N1	0.5 (4)
N4—N5—N6—C10	−0.1 (5)	C9—N1—C7—N2	−0.4 (4)
C6—C1—C2—C3	−1.5 (5)	C6—N1—C7—N2	175.1 (3)
C1—C2—C3—C4	1.5 (5)	C7—N2—C8—C9	−0.5 (4)
C1—C2—C3—C10	−179.1 (3)	N2—C8—C9—N1	0.3 (4)
C2—C3—C4—C5	−0.7 (5)	C7—N1—C9—C8	0.0 (4)
C10—C3—C4—C5	179.9 (3)	C6—N1—C9—C8	−175.5 (3)
C3—C4—C5—C6	−0.2 (5)	N5—N6—C10—N3	−0.1 (4)
C4—C5—C6—C1	0.2 (5)	N5—N6—C10—C3	179.3 (3)
C4—C5—C6—N1	−178.4 (3)	N4—N3—C10—N6	0.2 (4)
C2—C1—C6—C5	0.7 (5)	N4—N3—C10—C3	−179.2 (3)
C2—C1—C6—N1	179.2 (3)	C4—C3—C10—N6	10.2 (6)
C7—N1—C6—C5	39.0 (5)	C2—C3—C10—N6	−169.2 (4)
C9—N1—C6—C5	−146.3 (3)	C4—C3—C10—N3	−170.6 (3)
C7—N1—C6—C1	−139.6 (4)	C2—C3—C10—N3	10.0 (5)

### Hydrogen-bond geometry (Å, º)

**Table:**

*D*—H···*A*	*D*—H	H···*A*	*D*···*A*	*D*—H···*A*
N3—H3···N2^i^	0.86	1.93	2.751 (4)	158

Symmetry code: (i) −*x*+1, *y*−1/2, −*z*+1.
